# Computational Tissue Volume Reconstruction of a Peripheral Nerve Using High-Resolution Light-Microscopy and *Reconstruct*

**DOI:** 10.1371/journal.pone.0066191

**Published:** 2013-06-13

**Authors:** Mortimer Gierthmuehlen, Thomas M. Freiman, Kirsten Haastert-Talini, Alexandra Mueller, Jan Kaminsky, Thomas Stieglitz, Dennis T. T. Plachta

**Affiliations:** 1 Department of Neurosurgery, University Medical Center Freiburg, Freiburg, Germany; 2 Institute of Neuroanatomy, Hannover Medical School, Hannover, Germany; 3 Department of Neuropathology, University Medical Center Freiburg, Freiburg, Germany; 4 Department of Neurosurgery, St. Gertrauden Krankenhaus, Berlin, Germany; 5 Laboratory for Biomedical Microtechnology, Department of Microsystems Engineering, University of Freiburg, Freiburg, Germany; University of Arizona, United States of America

## Abstract

The development of neural cuff-electrodes requires several *in vivo* studies and revisions of the electrode design before the electrode is completely adapted to its target nerve. It is therefore favorable to simulate many of the steps involved in this process to reduce costs and animal testing. As the restoration of motor function is one of the most interesting applications of cuff-electrodes, the position and trajectories of myelinated fibers in the simulated nerve are important. In this paper, we investigate a method for building a precise neuroanatomical model of myelinated fibers in a peripheral nerve based on images obtained using high-resolution light microscopy. This anatomical model describes the first aim of our “Virtual workbench” project to establish a method for creating realistic neural simulation models based on image datasets. The imaging, processing, segmentation and technical limitations are described, and the steps involved in the transition into a simulation model are presented. The results showed that the position and trajectories of the myelinated axons were traced and virtualized using our technique, and small nerves could be reliably modeled based on of light microscopy images using low-cost OpenSource software and standard hardware. The anatomical model will be released to the scientific community.

## Introduction

The recent development of neuroprostheses generated different concepts for the interaction between electrodes and the peripheral nervous system [1], [2], [3]. These electrodes must offer the selective discrimination of the electrical activity inside the nerve and facilitate local fiber- or fascicle-specific stimulation without damaging neural integrity [4]. Multichannel cuff electrodes are one approach to this dilemma [5], [6]. These electrodes feature a large number of equally spaced contacts surrounding the nerve as an array of tripoles with a cathode in the middle of two anodes. These tripoles register the electrical activity on the surface of the nerve, but in contrast to invasive electrodes, the recorded signals must be processed using complex algorithms to find their sources inside the nerve [7], [8]. The electrodes have to match the nerve tissue, and the geometry of the electrode contacts has to be adjusted to the specific type of nerve and the nerve diameter. Therefore, cuff-electrode prototypes are mainly tested in vivo. The results are subsequently incorporated in new prototypes, which are again tested in animals. This procedure is both expensive and requires many laboratory animals before an electrode delivers valid results. Therefore, it would be favorable to move as much as possible of the electrode development into the computer and perform simulation studies to reduce costs and animal testing.

Currently, only simplified, idealized and mostly two-dimensional simulation models have been used [9], [10], [11]. These models are simple tubular constructions that neither reflects the natural anatomy of neural tissue nor the precise diameters of the axons. Instead, these models only simulate the position of the fibers in one section and neglect the fact that axons and fascicles show high mobility within the course of a nerve [12]. While cuff-electrodes capture the ENG (electroneurogram) and the mass activity of the myelinated axons underneath, it is literally impossible to record the single (SUA) or multi-unit activity (MUA) of individual axons. The shape and amplitude of external potentials strongly depend on the axon diameter, the trajectory and distance between the axons involved and the contacts with the electrode [13], [14]. This axonal mobility causes the surface potential of the nerve to be slightly different over the area that the recording electrodes integrate. Cuff-electrodes might also gently move forward and backward on the nerve resulting in changes in the long-term input/output function of the electrodes [15], [16] [17]. To simulate the input-output function of a multichannel cuff-electrode surrounding a nerve, the precise axonal diameters influencing the field-strength and conduction velocity are less important than their position and trajectory in a nerve segment, as the latter cannot be adjusted and would be lost in the simulation environment. For the development of robust recording algorithms, it is therefore essential to consider mobility-related changes in the surface potential to develop more realistic simulation models.

One of the most interesting applications for cuff-electrodes involves the restoration of motor function, e.g. a lost limb. In this scenario, the cuff-electrode surrounds a peripheral nerve in the proximal stump. The electrode records the ENG of this nerve and detects the activation of myelinated motor fibers. A computer transforms the signals into movements and controls the limb prosthesis. Although the number of small unmyelinated fibers might exceed the number of myelinated axons in a nerve, its surface potential is primarily influenced by myelinated fibers [13], relevant for motor restoration.

Based on the segmentation of single axons in a large confocal image set using *Reconstruct* software [18], this study addressed the following questions: (1) Is an image stack of consecutive light microscopy images sufficient to segment the myelinated portion of a peripheral nerve; (2) Could OpenSource software be used to create a realistic anatomical nerve model from this dataset; (3) Could this method also be applied for larger and longer nerve segments; and (4) How can the model be simplified for transformation into a simulation model without losing valuable precision?

## Materials and Methods

### Specimen Preparation and Histological Processing

This study was conducted in strict accordance with the recommendations in the Guide for the Care and Use of Laboratory Animals of the National Institutes of Health. The protocol was approved through the Ethics Committee of the University of Hannover (no. 4/51 according to animal law §4/3). All surgery was performed under pentobarbital anesthesia, and every effort was made to minimize suffering. A female Sprague-Dawley rat (200 g, Charles River, Germany) was sacrificed under deep anesthesia and the sciatic nerve with a small cutaneous nerve branch was resected through a dorsal incision at the thigh. The resected nerve was transferred to a fixative according to Karnovsky (2% Paraformaldehyde, 2.5% glutaraldehyde in 0.2 M sodium cacodylate buffer, pH 7.3 for 24 h) [19], [20], [21]. Afterwards, it was rinsed 3 times with 0.1 M sodium cacodylate buffer containing 7.5% sucrose prior to postfixation in 1% OsO4 for 1.5 h. Myelin staining was performed at 24 h in 1% potassium dichromate [22], followed by a 24-h incubation in 25% ethanol and an additional 24-h incubation in hematoxylin (0.5% in 70% ethanol). After dehydration, the tissue was embedded in EPON (SERVA Electrophoresis GmbH, Heidelberg, Germany).

The hardened specimen was loaded into an ultra-microtome (Leica Reichert Ultracut, Bensheim, Germany) and 0.8 µm thick serial slices were cut and mounted on glass slides (Langenbrinck SuperFrost Pro, Emmendingen, Germany) at 4–5 slices per slide. The cutting-level was axial to the nerve; however, in some cases, small cutting artifacts could not be avoided. Cover glasses were omitted, as they produce artifacts. After drying, the specimens were again stained with toluole (1 g toluidine-blue O in 49 ml Aq. dest +49 ml Aq. dest with 1 g di-sodium-tetraborate) and washed in distilled water. As this technique stains the myelin sheath of myelinated axons, unmyelinated fibers cannot be captured. The images were captured using a 20× objective on an Olympus BX61 microscope (Olympus, Hamburg, Germany). We used an Olympus E510 camera (10 megapixels single lens reflex, SLR), connected to the c-mount winding using an in-house-designed T-ring adapter. The focus was adjusted through the camera’s internal screen, and the camera was set to multispot automatic exposure. In this mode, the camera sensor uses different spots on the image to calculate the optimal exposure time instead of measuring only the center of the image. The resolution (2.6 pixel/µm) was sufficient for segmentation, and the cutaneous nerve branch was completely within the field of view; thus, no image stitching was necessary. We used Adobe Photoshop CS5 (*Adobe Photoshop CS5*, Adobe, Dublin, Ireland) to equalize the size of all images.

### Preprocessing

**Figure 1:**
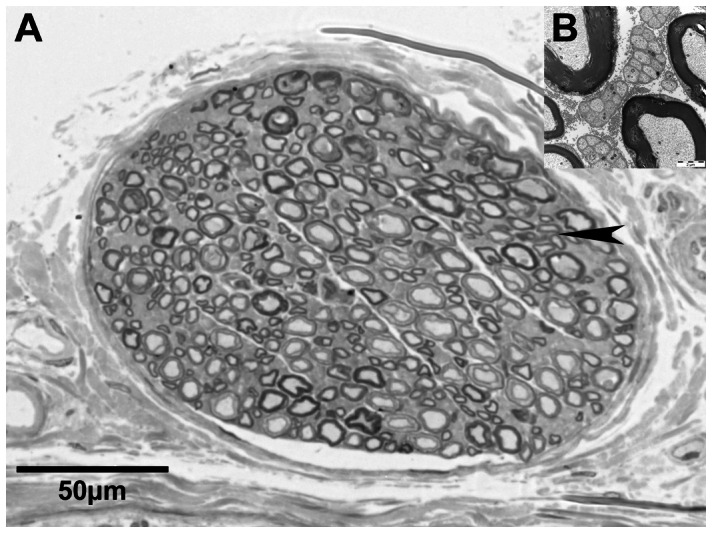
Microscopic overview of the segmented nerve. Light microscopic image of the small cutaneous nerve accompanying the rat sciatic nerve (A). Electron-microscopy of the area indicated by the arrowhead reveals umyelinated fibers as greyish matter between mylinated axons (B).

All images of the tissue slices obtained with the light microscope (Fig. 1) were aligned before segmentation. We used the *FiJi* “rigid – translate and rotate” function for alignment, as this function offers the full automatic alignment of a large number of images. Using this method, the images were not distorted to match each other, but rotated and moved to achieve the best fit. No manual adjustment was required. The alignment was confirmed after recording a video using the aligned image stack, which we optically assessed for inaccuracy. This evaluation did not reveal any alignment errors. After alignment, the surrounding area of the nerve was cut away using Xnview software (version 0.32, http://www.xnview.com), and the images were rotated 90° clockwise to achieve a better fit to the screen.

**Figure 2:**
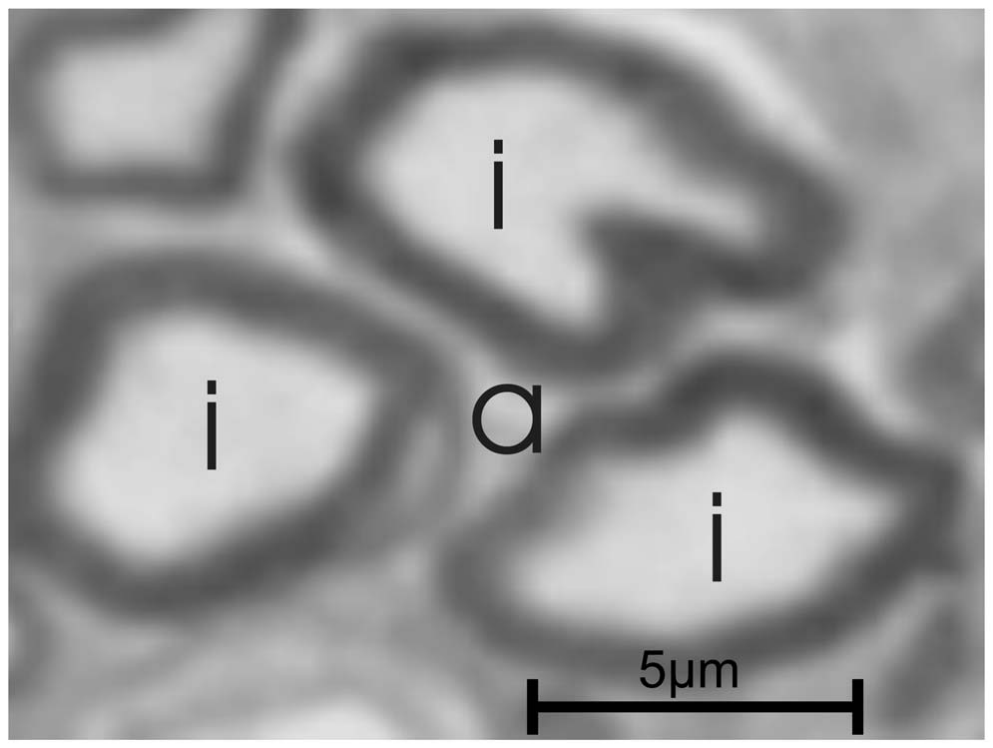
Touching axons creating a false axon. The fluorescent pictures are bicolored: the background is black and the structures are indicated with a specific color, e.g. red. In this figure, 3 axons (i) nearly touch, creating a semi-closed space (a), which might be interpreted as an additional axon using an automatic segmentation routine due to its color. The automatic segmentation of light microscopy images has therefore not only relies on color but also the shape features of the structure.

Generally, segmentation works best in black/white images, where the object is white and the background is black. *Reconstruct* features a tool called *Wildfire*, which uses a region-growing algorithm to detect axon borders. The user sets a seeding point and the software extends the point until certain criteria (e.g. difference in contrast, brightness or hue) are met. In our case, the inside of the axon was white, the sheath was black and the outside of the axon was white (Fig. 2). If the myelin sheath were always encased, the segmentation worked perfectly through propagation within the white inside of the axon. However, as open boundaries could not be avoided, the segmentation would also propagate outside the axon. Because the risk of an open boundary during the conversion from color to black/white is high, we converted the images to 8-bit gray scale using *Adobe Photoshop CS5.* The aligned images of the cutaneous nerve branch *(*625 images, representing 500 µm) were imported into *Reconstruct*. The *Wildfire* parameters were set to “Stop propagating if hue differs by 50”, “…if saturation differs by 50” and “…if brightness differs by 20”. All other options were set to default (*Automatically simplify “on”, Smooth using a moving-average filter length of “7”, Stop wildfire if area changes by 99%, Ignore regional wildfires of less than 0 square pixels)*. These parameters ensured that the *Wildfire* tool stopped growing over the boundaries of an axon and remained within the axon while propagating over the image stack. The thickness of the slices (which is the z-axis) was adjusted to 0.8 µm. To calibrate the x- and y-axes, the diameter of the nerve was microscopically measured (151.6 µm) and its length was defined in the menu *Calibrate*. The remaining sections of the domain were adjusted accordingly. This step was performed before any traces were drawn.

### The Segmentation Process

**Figure 3:**
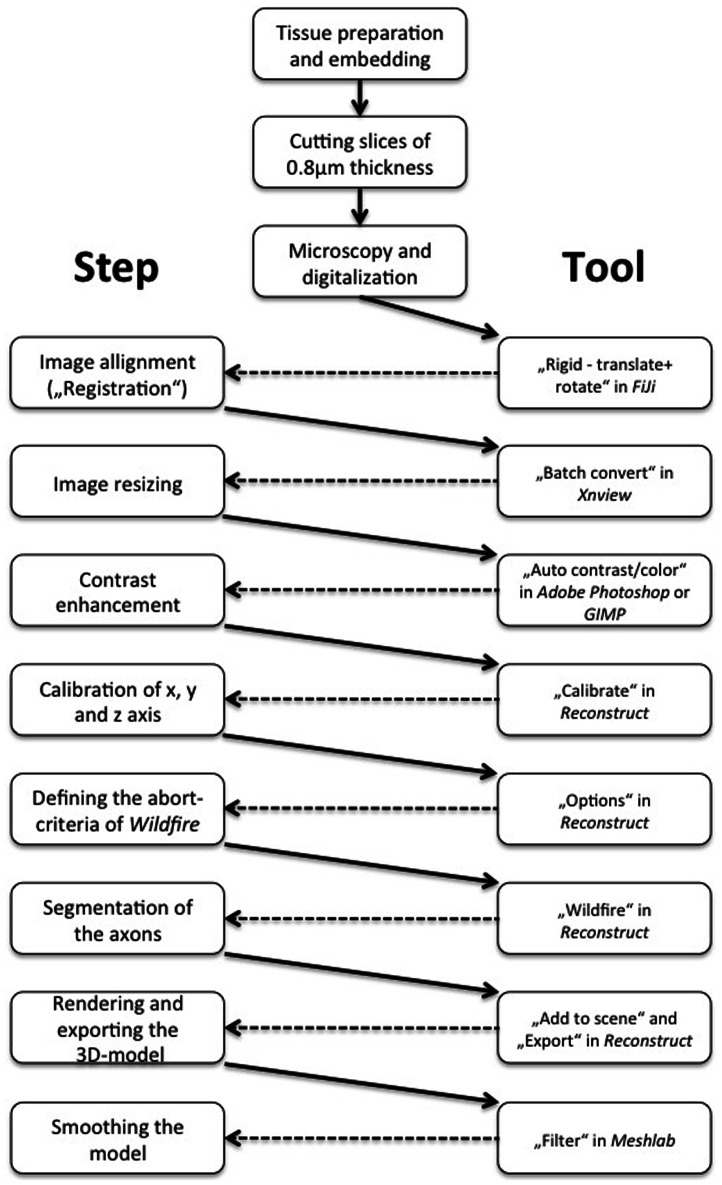
Workflow for segmentation including the software. Workflow from the nerve tissue to a virtual 3D model.

The seeding point of the *Wildfire* tool was set inside the axon on the first section. With 625 included slices, one traced axon consisted of 625 pieces called “domain1” on each slice, and at the end, these slices were referred to as “object1”. It is essential to ensure that all corresponding axons have the same name on the slices before the object is rendered; otherwise, the axons are assigned to other objects, resulting in rendering errors. If an axon is too small to have an inside that could be correctly filled by the *Wildfire* tool, then a circle can be drawn manually inside the axon. The workflow of the entire process is shown in Fig. 3.

### Analysis of the Segmentation

Semiautomatic tracing of individual axons through slices is an error-prone method. Many variables, such as artifacts during embedding, cutting, staining and particularly the low resolution of the digitized images, influence the quality of the segmentation. These variables cause the *Wildfire* algorithm associated with the growing region to fail during axon segmentation. Either no adjacent seeding point was observed, or *Wildfire* accidentally propagated outside of the axon. In both cases, the program stopped and waited for a user decision. These stop events were considered as tracing “abortions” and sufficient indicators of image quality. The variation of the single axon area from section to section during segmentation, called the fluctuation of the area, was another useful parameter. The *Reconstruct* software generated the inner cross-sectional area of each axon on each slide, but did not calculate the anatomical outer diameter of the axon, which includes the myelin sheath. The diameter, not the area or circumference, of axons is typically compared. However, this parameter could not be acquired directly from the images because the axons in slices are often oval-shaped and not perfectly round. To measure fluctuations in the area, we drew a circle with a congruent cross-sectional area in the inner cross-sectional area and measured the diameter of this circle. This idealized axon diameter was used only as a label for axon grouping in the statistical analysis.

**Figure 4:**
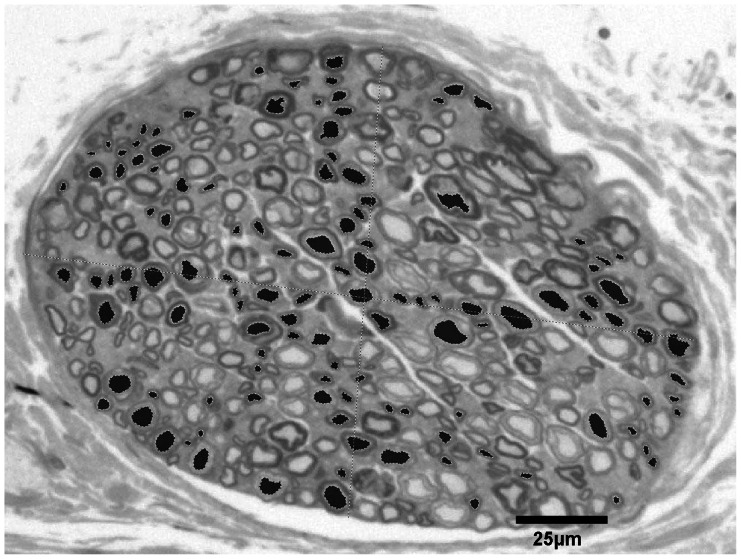
We selected 120 axons in a geometrical arrangement to cover all locations and sizes of the axons for the statistical analysis.

We expected the size of the axons and the z-resolution of the images to influence segmentation. We also determined whether the position of the axon inside the nerve was important. Therefore, we analyzed the correlations between (1) the tracing errors and (2) the fluctuation of the axon area during propagation based on a) the size of the axon (idealized axon diameter), b) the distance of the axon to the center of the nerve, and c) the z-resolution (inter-slice-distance, thickness of the slice). For this analysis, we randomly sampled 120 axons traced through a 25-µm segment of the nerve (see Fig. 4).

## Results

We identified three different sources of error: (1) Permanent systematic errors, such as the shrinkage of embedded material, (2) spontaneous systematic errors, e.g., the axons outside of the ideal cutting plane (90°), and (3) the resolution of the image. The missing outer diameter of the myelinated axons can be addressed in the simulation environment using the g-ratio [23]. In addition, the tissue shrinkage can be addressed using a compensation factor in the modeling environment. The spontaneous systematic errors and the image resolution were statistically analyzed.

### Normalization of the Data

**Figure 5:**
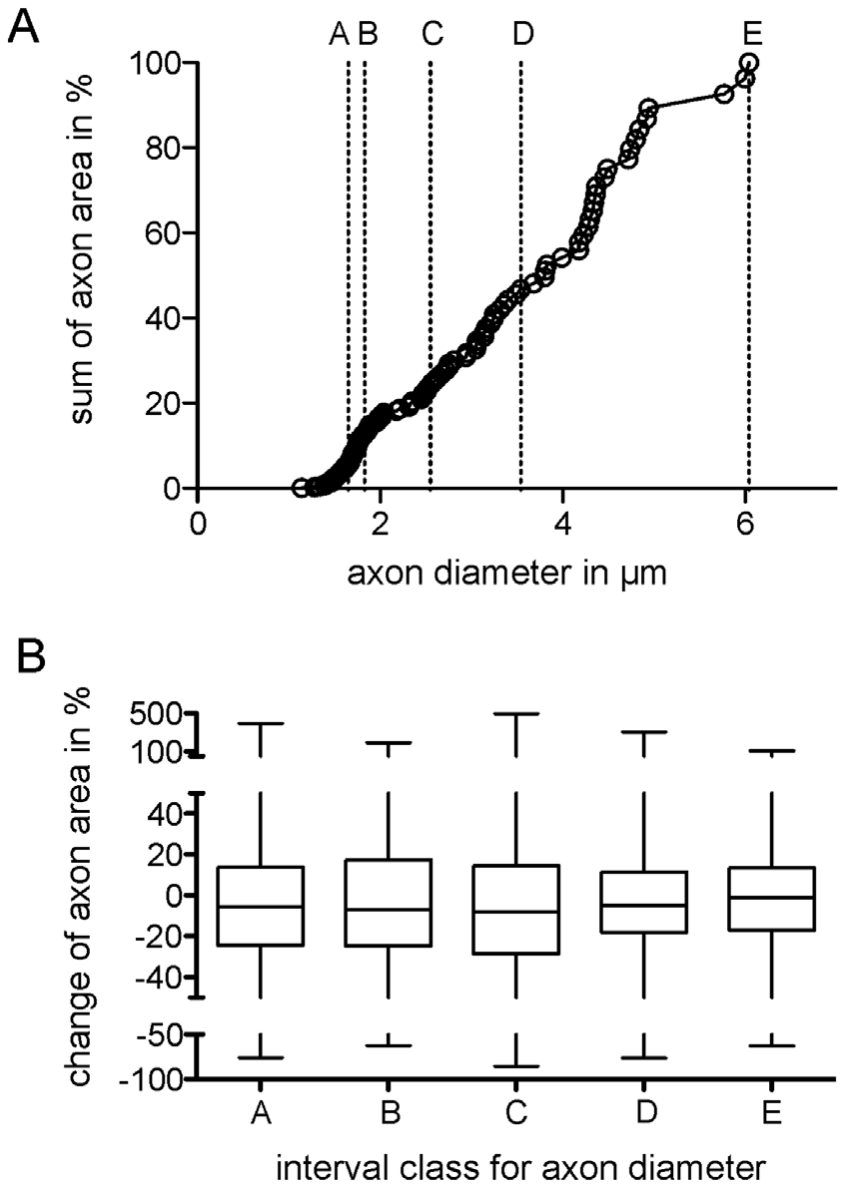
Comparison of the classes. *A*: Distribution of the idealized diameters of axons with respect to the sum of all measured inner cross-sectional axonal areas in %. The classes A to E contain 24 axons each, within the following diameter intervals: (A) 1.14 µm to 1.65 µm, B) 1.66 µm to 1.83 µm, C) 1.84 µm to 2.55 µm, D) 2.56 µm to 3.54 µm, E) 3.55 to 6.04 µm. *B*: Median and quartiles for each group were calculated on the basis of the individual derivations from the mean area within the individual axons. The narrowest quartiles were observed in the largest axon classes. In the pooled approach, the five classes were significantly different from each other (Kruskal-Wallis test, p = 0.0006). Note that while all classes are significantly different, the larger axons have diameter variation through the tracing of 32 slices.

As previously discussed, the only measured variables in this experimental work were the axonal areas, the number of tracing errors and the distance of the axons to the center of the nerve. For comparative reasons, we examined the distribution of the average axon diameter (idealized from the mean areas of individual axons) in the sample. The mean diameter of every axon was obtained from the pooled axons in every slice, as described above. The cumulative plot in Fig. 5A illustrates the axon distribution observed in the sample. The majority of axons had an average diameter between 1.3 and 2.3 µm, with a minimum of 1.14 µm and a maximum at 6.04 µm and no compensation associated with shrinkage or the g-ratio. Next, we determined whether larger axons (with larger mean areas and thereby larger mean diameters) showed similar fluctuations in the area over consecutive slices compared with smaller axons. As a precondition for the statistical analysis, we constructed five groups with identical N ( = 24), covering different axonal sizes (diameters) (see groups A to E in fig. 5A). Because the area of every axon in the slice is known, we compared the variation of this parameter around the mean. This calculation was performed for each axon, and the parameters (max. and min. deviation from the mean) were pooled for the 24 axons belonging to one group (A through E) in the sample. As shown in Fig. 5 (B), we chose the measured inner cross-sectional area as the basis for the calculation. Because the area increases non-linearly (quadratically), larger axons show disproportionally larger changes in the area fluctuation across consecutive slices compared with smaller axons. For a comparison between large and small axons, the non-linear increase in the area was normalized to avoid invalid statistical results. For the area normalization, the mean area (for every axon individually) was set to 100%, and deviations from this mean, observed in every slice, were calculated as a percentage, ruling out inherent non-linearity. The deviation results of the grouped axons were pooled. The box plot in Fig. 5 (B) shows the median and quartiles for each group, with the maximum and minimum whiskers. Every box comprised 768 data points (24 axons×32 slices at 0.8 µm inter-slice interval). The medians were all close to zero %, and the quartiles were symmetrical, indicating that there were no major asymmetric biases. The narrowest quartiles were detected in the largest axon classes, suggesting that smaller axons showed a larger fluctuation in the area than larger axons. Therefore, we assessed the similarity of these five groups. Based on a pooled approach, the five groups were significantly different from each other (Kruskal-Wallis test, p = 0.0006) with respect to standardized area fluctuations. However, not all classes remained significantly different from using an individualized approach (Welch t-test, Table 1). The two groups with the smallest axons (and also diameter) showed proportionally larger fluctuations in the inner area compared with the three groups with larger axons.

**Table 1:** Results of the individual comparison of the normalized area fluctuation for grouped axons of given diameter intervals (Welch t-test).

Compared classes:	Significance at:
A–B	not
A–C	p 0.0001
A–D	p 0.0001
A–E	p 0.0001
B–C	p 0.0001
B–D	p 0.0117
B–E	p 0.0001
C–D	p 0.0001
C–E	p 0.0001
D–E	p 0.0001

The diameter intervals from A to E are shown in Fig. 5.

### Influence of the z-resolution

The standard z-resolution was 0.8 µm, defined by the thickness of slices obtained using a microtome. We determined whether using every second or every third slice (thus increasing the z-interval to 1.6 µm or 2.4 µm, respectively) would accelerate segmentation or increase the tracing error rate. In this approach, the number of tracing abortions reflects the robustness of the region-growing algorithm *Wildfire*. However, increasing the slice-interval accelerates segmentation when the number of tracing abortions, which require manual interaction, does not increase, thereby effectively reducing the segmentation speed. We compared the relationship between the abortion rates and the inter-slice intervals. With 0.8 µm inter-slice intervals, 207 abortions occurred during the segmentation of 120 sample axons over 32 slices (0.054 abortion/axon/slice). At a 1.6 µm inter-slice interval, the abortion rate increased to 277 tracing abortions over 16 slices (0.14 abortion/axon/slice) and with a 2.4-µm inter-slice interval, the abortion rate further increased to 300 abortions over 11 slices (0.23 abortion/axon/slice). As expected, the inter-slice-intervals strongly influenced the abortion rates in a size-dependent manner.

### Influence of the Axon Size

**Figure 6:**
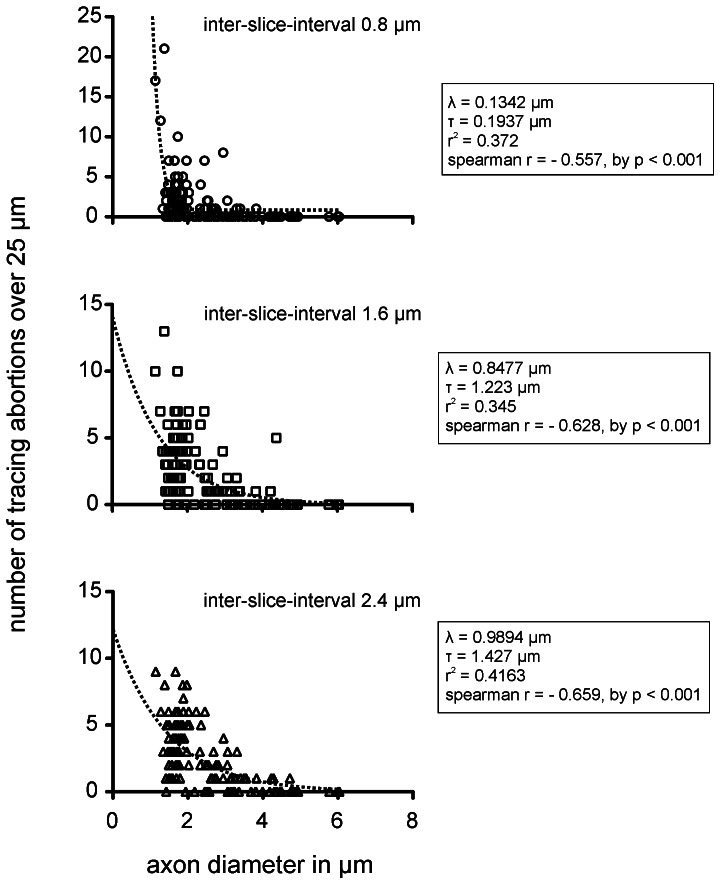
Absolute number of tracing abortions during segmentation in correlation to the inter-slice intervals. Absolute number of aborted segmentations, as a function of the (idealized) axon diameter, for the three different inter-slice intervals (top: 0.8 µm, middle: 1.6 µm, bottom: 2.4 µm). The plots contain exponential fits (one phase decay). Due to heteroscedasticity in the scatter, we used Spearman’s rank correlation coefficient to quantify the slope of the correlation between tracing abortions and axon diameters. Note: The absolute number of tracing abortions correlates negatively with the axon diameter and the steepness of the slope of the non-linear correlation increases with the inter-slice interval. The increasing inter-slice interval greatly affects the abortion rate (see λ and τ of exponential fit) for axons with diameters below 4 µm (inner cross-sectional areas below 12.57 µm^2^).

We next determined whether the axon size (using the idealized diameter as a label) affects the abortion rates. This relationship of three different inter-slice intervals (a = 0.8 µm, b = 1.6 µm, c = 2.4 µm) is shown in Fig. 6. Because the distributions of the tracing abortions associated with the axon diameter (obtained from the measured mean axon area) showed strong heteroscedasticity, we calculated the non-linear single-phase fit and the Spearman’s rank correlation coefficient to quantify the slope of the correlation. The scatter plots and the statistical values illustrate that, independent of the tracing abortion rates in the inter-slice intervals, the axon size significantly influences the occurrence of tracing abortions. Small axons generated the majority of all tracing abortions, resulting in a strong negative slope for all Spearman’s r-values. Axons with an idealized diameters below 4 µm showed the highest abortion rates.

### Influence of the Axon Position in the Nerve

**Figure 7:**
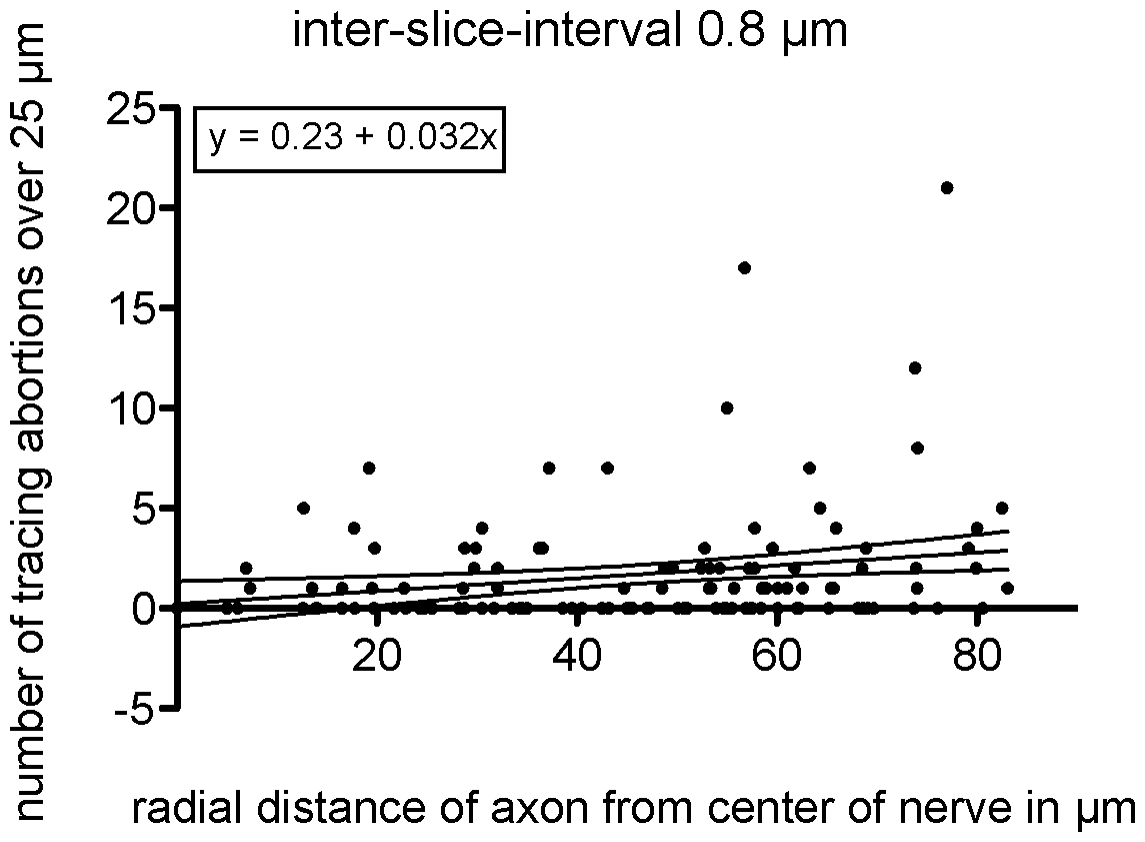
Number of abortions with respect to the axon position. Using the radial distance of the axons from the center of the nerve as the abscissa against the abortions that occurred for each axon, the linear correlation shows a positive slope. Although the correlation coefficient is weak, the positive slope is significantly different from zero (p = 0.0195).

We also determined whether the position of the axon near the center or the surface of the nerve affected the abortion rate during segmentation. In the previous chapter, we observed a strong correlation between the axon sizes and tracing abortion rate, with the majority of the tracing abortions observed in small axons. Accordingly, we first determined whether the axon size is correlated with the distance from the center of the nerve. To rule out this bias and thus false positive results, we calculated Spearman’s R test to verify any significant correlation between the axon size and the distance from the nerve center. We did not observe any correlation (r^2^ = 0.0092, p = 0.9198). In addition, a linear regression on the axon diameter across the axon distance from the center showed an almost horizontal fit (r = 0.003, slope = −0.0003, not significant from 0), which further supports this conclusion. An analysis of the tracing abortion rate, as a function of the axonal distance from the nerve center, revealed a slight positive relationship (Fig. 7) between the distance from the center and the abortion rate. Although the correlation coefficient was low due to the scatter, the result was significantly different from zero (p = 0.0195).

Thus, axons of different sizes were distributed homogenously across the distance from the nerve center to the periphery, and the position of an axon inside the nerve slightly influenced the abortion rate. However, inter-slice interval and axon size were the most critical parameters.

### Rendering

In the final model, 625 slices of a 0.5 mm peripheral nerve with an inter-slice interval of 0.8 µm were used. The rendering time showed a linear correlation with the increasing number of included axons and slices. *Reconstruct* 1.1.0.1 did not benefit from a multicore processor, but relied on only one or two cores at a time. The model was stored as a VRML2.0-model, imported in Meshlab (version 1.3.0, http://sourceforge.net/) for further processing.

### Smoothing

**Figure 8:**
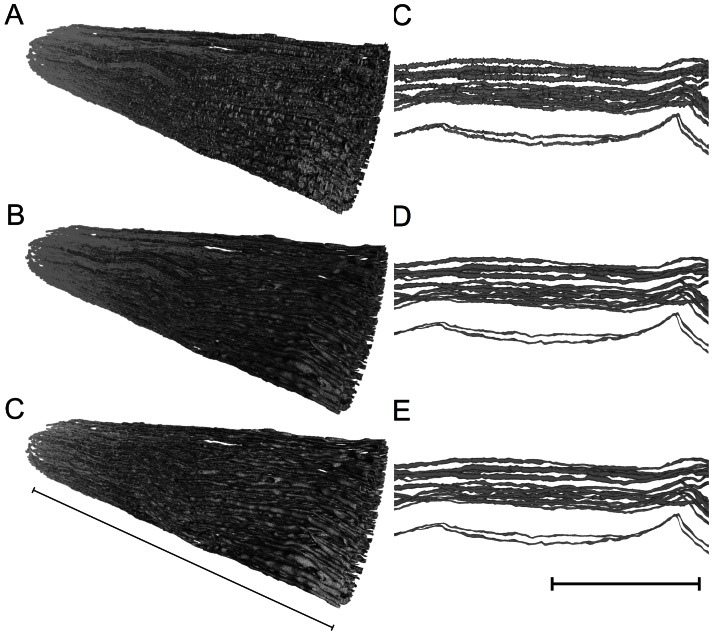
Final smoothing of the model to reduce file size. *A to C* Different stages of model processing in Meshlab showing the nerve in isometric view (A = unprocessed model from *Reconstruct*, B = smoothed model HC Laplacian smoothing, C = after smoothing and vertex reduction using “Quadratic based edge collapse strategy”). *A to D to F* The same processing steps, showing a horizontal zoomed view of only a few axons of the nerve model. The processing of D corresponds to A, E to B and F to C. Details regarding the filter algorithms are discussed in the text. Note that the smoothing only mildly reduced the thickness of the axon diameter and the reduction of vertices from 4,296,603 to 1,666,224 neither alters the smoothed surface nor the results in new artifacts. Left scale bar indicates 0.5 mm, right scale bar indicates 80 µm.

The 3D model from *Reconstruct* suffered from interpolation during the rendering process, suggesting that the surface of the axons was artificially detailed (see Fig. 8, top). The over-detailed surface was reduced to the essential elements without losing important anatomical features, such as the diameter and the trajectories of the individual axons, to transfer this model into a simulation environment using a standard computer. Therefore, two steps were performed: A) a smoothing to level the artificially detailed surface of the axon without reducing the axon diameter, and B) a reduction of the vertices [9]. The vertices are the positions that connect the edges in a polygonal mesh. Meshlab is an OpenSource software program for building, visualizing and editing polygonal meshes. We used this program with triangle faces based on three vertices connected by three edges. This analysis offers several algorithms to perform these modifications, even with large data sets, such as our nerve model. Our original model consisted of 4,296,603 vertices and 8,580,661 faces (elements), resulting in a file of 630 MB. To initiate this file in Meshlab, at least 8 GB of RAM and the 64-bit version of Meshlab were needed, as the file consumed up to 4 GB of RAM. The application easily consumed 7 GB of RAM on a Windows 7 PC during filter processing. Meshlab offers many options to A) smooth surfaces and B) reduce the number of vertices; however, not all options conserve the original shape and volume of the axon. The two most appropriate options were “HC Laplacian smooth” for smoothing and the “Quadratic edge collapse decimation” for simplification (reduction of vertices). The HC Laplacian smooth feature calculated the average position for every vertex in relation to its neighbors and compensated for the shrinkage of the axon diameter or the deformation of the smoothed surface. Details on the filter are provided in the original work of Vollmer, Mencl and Müller [24]. The Quadratic edge collapse decimation option merged the adjacent vertices. The smoothing filter did not allow any further settings or adjustments. The simplification filter of maximal 0.4 (%) resulted in a reduction of vertices to 38%. Higher reductions resulted in new artifacts and changed the topology of the axons. The raw models, compressed as the ZIP-file “models_vrml_ply.zip”, can be downloaded under http://sdrv.ms/105uVdk.

## Discussion

### (1) Is an Image Stack of Consecutive Light Microscopy Images Sufficient to Segment the Myelinated Portion of a Peripheral Nerve?

Three-dimensional models are used to visualize structures and conduct simulation studies to enhance the current understanding of the function of the biological objects. The reconstruction of these objects, called segmentation, is based on 3D imaging methods, such as MRI [25] or computer tomography volumes [26]. These datasets are primarily isotropic, suggesting that the x, y and z resolution are the same. Because the tissue is imaged in situ without cutting it into slices, the images do not need alignment. Magnetic resonance imaging (MRI) can be used to transform a long nerve segment into an isovoxel 3D dataset without further alignment. However, the MRI resolution, even in high-tesla machines, is limited to a few hundred micrometers, which is not sufficient for the visualization of microscopic structures, such as axons. The same limitations apply for computer tomography. Transmission electron microscopy provides sufficient resolution, but tissue preparation is too complex to perform manually using several hundred sections. Therefore, these imaging techniques did not qualify for our intended purpose.

Fluorescence [27], electron [28] and light [29] microscopy visualize small structures, such as axons, but only produce 2D images. Aligning and stacking consecutive 2D microscopic images can generate a 3D dataset. However, cutting, imaging and aligning the slices is time consuming. The automated acquisition of a true 3D dataset would therefore be favorable, particularly if longer (several millimeters) and bigger (several thousand axons) nerves are imaged. Advanced confocal, fluorescence microscopes produce 3D datasets using an excitation beam and optical focus through specimen of up to 200 µm in thickness. Confocal imaging relies on fluorescent staining techniques. A contrast dye is typically applied to the nerve in vivo, requiring surgery prior to sacrificing the animal. An additional disadvantage of this staining is that not every axon absorbs the contrast dye, and therefore remains invisible to the microscope. In addition, fluorescent staining is more complex and time consuming than light microscopy, post-mortem cryosection tissue preparation alters the anatomy and cutting artifacts of cryosections are common. In practice, the thickness of the specimen, i.e., the length of the nerve segment, is limited to approximately 200 µm.

The Lichtman group [18] described the reconstruction of neural processes using confocal imaging and *Reconstruct*. Our study was based on this work, but there are several differences between our approach and that of the previous study. Lu used confocal microscopy in a Brainbow mouse. This transgenic animal expresses a fluorescent dye and needs no further in vivo staining [30]. We want to adapt the cuff-electrode to larger animals, starting with the rat, but no Brainbow rat has been developed yet. Secondly, Lichtman traces five axons over an area of approximately 30×30 µm. In our study, we traced 338 axons over an area approximately 150×150 µm, resulting in a 25 times larger area. Even with five axons, Lu performed image stitching and produced 20.000 images, corresponding to 500.000 images in our scale.

In addition, we attached a 10-megapixel camera to the light microscope, facilitating the capture of a single image of the entire nerve with decent resolution to trace the myelinated fibers. A confocal microscope is equipped with a camera, which is directly controlled by the microscope computer. In our study, the cameras had resolutions between three and five megapixels, which is not sufficient for taking a single picture of the nerve and identify single axons. Therefore, image stitching is necessary. Moreover, we want to simulate larger nerves in rats and larger animals. However based on the reasons described above, we believe that confocal microscopy is not suitable for the segmentation of long segments of larger nerves.

There were several advantages to labeling and light microscopy imaging. The nerve could easily be harvested and incubated from a sacrificed animal, which preserved the anatomy. The sections were thin (0.8 µm) and easy to acquire using high resolution. The block mounted on the microtome was only limited in the z-axis, and a standard light microscope was sufficient for imaging. Because a standard camera with a 10-megapixel resolution was connected to the microscope, we could use a low (20×) objective to keep the whole nerve in the field of view and identify the myelinated axons. Image stitching was not necessary, but could easily be performed using OpenSource ImageJ software version *FiJi* (http//fiji.sc) in larger objects. This software is also capable of performing image alignment. We stained the myelin sheath using the Karnovsky technique. In preliminary experiments with a rat sciatic nerve, we experienced central fixation artifacts at approximately 250 µm from the stump when simple paraformaldehyde (PFA) fixation was applied without myelin staining. The fixing agent did not penetrate the sciatic nerve more than 200 µm –250 µm radially when the rat was perfused transcardially with PFA, potentially reflecting the tight myelin sheaths of the rat peripheral nervous system [31]. We also used this staining method for the cutaneous nerve branch, although this structure was much smaller. A disadvantage of this staining and imaging technique is the lack of sensitivity to unmyelinated axons, which are not stained and cannot be detected. These fibers are also extremely small (0.5–1.4 µm in diameter [32]) and often collapsed, with an inside area below the resolution of our imaging technique. As investigated in electron microscopic studies [33] and seen in Fig. 1B, the fibers are detected as “grayish matter” between the myelinated axons. The suitability of the resulting nerve model for simulation studies depends on the application. If the motoric portion of a nerve, consisting of only myelinated A and B fibers [34], is investigated, for the development of peripheral nerve electrodes for the restoration of motor function, then a nerve model consisting of only myelinated fibers is sufficient. These fibers are much larger than unmyelinated axons and dominate the compound surface potential [13], [14] particularly when several hundreds or thousands of fibers are synchronized during motor activation. Neither the staining nor the imaging technique presented is sufficient to study unmyelinated fibers.

A promising alternative for the segmentation of longer and bigger nerve segments is the automated Serial Block Face Scanning Electron Microscopy (SBFSEM) [35], [36], [37]. In this method, the specimen is mounted and the face of the block is scanned using an electron microscope before the slice is cut with the microtome. As scanning and cutting can be automated, a large volume of images can be acquired overnight without the need for alignment. In addition, the electron microscope provides a high XY-resolution of 0.2 µm, and the slice thickness can also be as low as 0.2 µm, to create a true 3D isovoxel dataset. However, whether SBFSEM detects and follows unmyelinated axons remains the subject of further studies, resulting in more nerve models.

### (2) Can OpenSource Software be used to Create a Realistic Anatomical Nerve Model from this Dataset?

Segmentation often relies on the region-growing technique. In this method, the user sets one or several seeding points and the algorithm starts growing until the boundaries of the structure are reached. The stopping criteria have to be adjusted individually and the structures should have a high contrast to the background for recognition. Despite the fact that there are several commercial and OpenSource software packages available (Table 2), these programs either cannot propagate a manually defined region over a stack of images, rely on a medical data format (e.g. DICOM) or only accept genuine isovoxel 3D datasets. In addition, the number of 338 individual objects (axons) exceeds the capacity of most programs. In the end, most of the available commercial alternatives are expensive. The OpenSource program *Reconstruct* (version 1.1.0.1) was the only software that could handle propagate several hundred objects over a large volume of slices. This program does not rely on isovoxel datasets and provides several ways to perform manual adjustments (http://synapses.clm.utexas.edu/tools/reconstruct/reconstruct.stm). *Reconstruct* has previously been used to segment neural processes from a large fluorescent image stack containing 20,000 images [18]. Its region-growing tool *Wildfire* facilitates the definition of abort criteria (e.g. contrast, hue, brightness) and propagates the manually selected structure through the image stack until the abort criteria are met. The preprocessing of the original images using Adobe Photoshop could also be addressed using the open source software GIMP (http://www.gimp.org). The segmentation precision significantly depended on the size of the axon. As shown in Fig. 6, axons with diameters below 4 µm (with inner cross-sectional areas below 12.57 µm^2^) delivered the highest abortion rates. Smaller axons also showed significantly larger area fluctuations than larger axons (Tab. 1 and Fig. 5B). We have not learned whether this latter effect is an inherent effect of smaller axons or, as we suspect, reflects the limited resolution of our optical system. As a rule of thumb, a resolution of 10 pixels per axon (if not smaller than 1 µm) should be used to avoid rendering abortions. The inter-slice interval strongly affects the tracing quality, and the tracing abortion rates significantly correlate with the increasing inter-slice distance. We recommend using an inter-slice interval range of 0.8 to 1.6 µm. The relative position of the axon to the center of the nerve only mildly affects the reconstruction quality independent from the distribution of the axon size across the distance from the nerve center, as this distribution was homogeneous, potentially reflecting imaging artifacts due to the handling and cutting of the slices. Better embedding technologies might result in less cutting artifacts.

**Table 2:** Commercial and OpenSource software for the segmentation of biological and medical datasets.

Name	Company	URL	License
ScanIP	Simpleware	http://www.simpleware.com/software/scanip/	**Commercial**
Amira	Amira	http://www.amira.com	
Bioquant	Bioquant	http://www.bioquant.com	
ImagePro	Mediacy	http://www.mediacy.com	
ArivisBrowser	Arivis	http://www.arivis.de	
EM3D	Stanford	http://em3d.stanford.edu	**OpenSource**
TrakEM2	University of Zurich	http://www.ini.uzh.ch/~acardona/trakem2.html	
Slicer	Slicer	http://www.slicer.org	
FreeSurfer	Harvard University	http://surfer.nmr.mgh.harvard.edu	
VANO	Vano	http://vano.cellexplorer.org/Home	
Imagesurfer	University of North Carolina	http://imagesurfer.cs.unc.edu	
KNOSSOS	Max-Planck Institute for MedicalResearch Heidelberg	http://www.knossostool.org	
Ilastik	HCI: Heidelberg Laboratory forImage Processing	http://www.ilastik.org	

Our 3D model represents the position and trajectories of myelinated fibers and is suitable for the simulation of mass signals or ENGs of the motoric neural portion. In addition, the selectivity of stimulation through a virtual electrode wrapped around the nerve model can be investigated. For simulation of nerves, including unmyelinated fibers, the histological processing has to be adapted to reach these axons. The electrical properties (for instance the type and speed of action potential propagation) can then be adjusted in the simulation environment. If a model for the simulation of *penetrating* nerve electrodes is necessary, then the axon diameter must be precisely reconstructed. Thus, our method would not be suitable.

### Could this Method also be Applied for Larger and Longer Nerve Segments?

The region growing technique in *Reconstruct* relied on manual intervention of the user and was extremely time consuming, as the 500-µm long nerve segment comprised 338 axons. Segmentation through the entire stack took 5–10 minutes per axon; thus, approximately 60 hours of segmentation was necessary. For larger and longer nerve segments, alternative algorithms, such as template matching, edge detection, active contours, zonal graphs and neural networks [38] should be investigated. These algorithms are often programmed for a highly specialized tasks, such as the reconstruction of a grain [39] or the lamina of vessels [40], and depend on the recognition of certain patterns [29]. The recognition of axons is typically used for the automated analysis of axon numbers on a single histological image [41], and many commercially available microscopic software packages provide a basic axon-counting function and the segmentation of single dendrites through a 3D dataset. The semi-automated 3D reconstruction of a small number of axons through electron microscopic image sets has recently been examined [42], [43]. If the segmentation of larger nerves is necessary in the future, intelligent pattern-recognition algorithms should be programmed to detect axons in an image stack to reduce manual intervention [40].

### How can the Model be Simplified for Transformation into a Simulation Model without Losing Valuable Precision?

Segmentation produces a detailed axon surface. The over-detailed surfaces increase the number of vertices and demand high resolution meshing, which also results in higher calculation power for simulation. The mesh and thus the surface should be simplified if the simulation is performed on standard workstations. We also detected problems with the high resolution of surfaces during simulation. If the surfaces of two adjacent axons are too close, some of the vertices of the surface of one axon could become incorporated into the surface of the other axon. If overlap occurs, the FEM simulation fails due to a “short-cut”. The smoothing process described above was not sufficient to eliminate all overlapping errors between borders of adjacent axons. However, the absolute numbers of overlapping errors in our model dropped from 6.815 prior to smoothing to 213 after smoothing. To what degree the reduction of the vertices to 38% will reduce the quality of our model in the simulation will be subject to an analysis of future studies.

In contrast, the size and, more importantly, the position of an axon should be preserved during smoothing. In a functional model, the diameter of an axon influences the signal propagation speed, signal strength and the sensitivity of the axon to stimulation charges. Accuracy in calculating the axon diameter is therefore important, but because these three parameters can be independently addressed in the simulation environment, the anatomical surface is not as important for the model as the relative position of the axon within the nerve. This relative position, the trajectory of the axon, is the most crucial parameter for our functional model in which surface potentials are calculated, which is hardly affected by processing errors. Once the cuff surrounds a nerve, its contacts form a rigid geometrical grid [8], [44]. The spacing of the contacts directly impacts both the recorded signals and the selectivity of the stimulation using multichannel electrodes [45]. To construct a test bench to design the cuff electrode, the preservation of the trajectory of individual axons is necessary. This trajectory is the most valuable parameter, as it cannot be regained or parameterized once the model is transferred to the simulation environment. Thus, our model provides the preservation of the individual axon trajectory over all existing functional nerve models that feature individual axons in the z-axis as static tubes.

### Conclusions

Small nerves not exceeding a hundred myelinated axons and a few hundred micrometers in length can be visualized by segmenting a stack of light-microscopic images with the OpenSource software *Reconstruct*. Larger and longer nerve segments and unmyelinated fibers are not suitable for this imaging technique or segmentation due to the high degree of manual interaction. In addition, unmyelinated fibers are not captured through staining and are too small to be reliably imaged using light microscopy. In this study, we present a 500-µm model of a small peripheral nerve containing 338 myelinated axons. This model is the first to preserve the individual trajectories of every myelinated axon inside the nerve, which play an important role in the development of realistic simulation models. OpenSource programs can be used for segmentation and processing on standard computer components, keeping the costs for the project to a minimum. The presented anatomical nerve will be converted into a physiological model in the environment COMSOL to simulate the nerve excitation and propagation properties and the distribution of the electrical field on the surface of the nerve. The raw models, compressed as a ZIP-file named “models_vrml_ply.zip”, can be downloaded under http://sdrv.ms/105uVdk.
